# Characterization of the Fungal Microbiota (Mycobiome) in Healthy and Dandruff-Afflicted Human Scalps

**DOI:** 10.1371/journal.pone.0032847

**Published:** 2012-02-29

**Authors:** Hee Kuk Park, Myung-Ho Ha, Sang-Gue Park, Myeung Nam Kim, Beom Joon Kim, Wonyong Kim

**Affiliations:** 1 Department of Microbiology, Chung-Ang University, Seoul, South Korea; 2 Department of Dermatology, College of Medicine, Chung-Ang University, Seoul, South Korea; 3 Department of Applied Statistics, Faculty of Business and Economics, Chung-Ang University, Seoul, South Korea; Argonne National Laboratory, United States of America

## Abstract

The human scalp harbors a vast community of microbial mutualists, the composition of which is difficult to elucidate as many of the microorganisms are not culturable using current culture techniques. Dandruff, a common scalp disorder, is known as a causative factor of a mild seborrheic dermatitis as well as pityriasis versicolor, seborrheic dermatitis, and atopic dermatitis. Lipophilic yeast *Malassezia* is widely accepted to play a role in dandruff, but relatively few comprehensive studies have been reported. In order to investigate fungal biota and genetic resources of dandruff, we amplified the 26S rRNA gene from samples of healthy scalps and dandruff-afflicted scalps. The sequences were analyzed by a high throughput method using a GS-FLX 454 pyrosequencer. Of the 74,811 total sequence reads, Basidiomycota (*Filobasidium* spp.) was the most common phylum associated with dandruff. In contrast, Ascomycota (*Acremonium* spp.) was common in the healthy scalps. Our results elucidate the distribution of fungal communities associated with dandruff and provide new avenues for the potential prevention and treatment of dandruff.

## Introduction

Unlike classical seborrhoeic dermatitis, dandruff is a non-inflammatory condition of the scalp that is characterized by scaling and is considered to be a form of mild seborrheic dermatitis. Dandruff is a common scalp disorder affecting almost half of the postpubertal population regardless of ethnicity and gender and has several putative causes including non-microbial and microbial factors [1]–[3]. The potential non-microbial causes for dandruff are excessive exposure to sunlight, minimal irritation of the scalp due to over shampooing, frequent combing, use of certain cosmetic products, and exposure to dust and dirt, although experimental evidence is lacking[1], [4], [5]. The microbial etiopathology that is most widely accepted is the presence of a lipophilic yeast belonging to the genus *Malassezia* [1], [6]. The scalp has a biotic community of which the known components are *Staphylococcus* spp., *Propionibacterium* spp., and *Malassezia* spp. [7]. On the dandruff-afflicted scalp, the levels of *Malassezia* increase by 1.5 to 2 times their normal level [8]. *Malassezia* species are also known as a causative factor in pityriasis versicolor, seborrheic dermatitis (SD), and atopic dermatitis (AD) [9], [10]. *Malassezia* consists of at least seven species, each of which has specific ecological, biochemical, and genetic characteristics. Some of the *Malassezia* species (*M. furfur*, *M gobosa*, and *M. restricta*) are associated with various human infections but the pathological role of each species is not fully understood [10]–[13].

Recent advances in studies of metagenomic approaches, such as mass pyrosequencing enhance insight into microbial community structure and function. The fast-growing field of metagenomics provides knowledge regarding the genomes of environmental microbes and entire microbial communities. Metagenomics has been studied in different human tissues such as the gut, skin, and oral cavity [14]–[16]. Such research provides a conceptual and technical base for the species and community data to form a model framework, thus revealing the functions and interactions between networks of species in a multispecies environment. Knowledge of the frequency and distribution of potential microbial etiological agents, such as possible pathogenic fungi on the human scalp, is important for understanding the epidemiological cycle of these fungi and their impact on scalp health. Understanding the specific relationships between *Malassezia* species or between *Malassezia* and other fungus species may be useful in developing a strategy for treating dandruff.

In this study, we investigated the fungal communities associated with dandruff on the human scalp using the GS-FLX Titanium sequencer (Roche), massively parallel ribosomal RNA gene amplicon pyrotag sequencing. We amplified the 26S rRNA gene from fungal species collected from basal (healthy) scalp samples and dandruff-afflicted scalp samples. To our knowledge, this is the first study of the mycobiome present on both basal scalps and dandruff-afflicted scalps.

## Results

### Sequence reads of 26S rRNA gene

A total of 74,811 sequence reads of 26S rRNA gene were obtained using the Genome Sequencer FLX Titanium platform (Table 1). Each sample has similar patterns of size distribution, and 13–19% of the sequences were smaller than 300 bp. Sequences that were too short to be used to identify species and that had no significant similarity found in the public database were removed from the analysis. Thus, from a total of 66,212 (88%) sequence reads including 10735,10029, 12119, 9614, 9092, 7589 and 7034 reads from healthy scalp samples and dandruff-afflicted scalp samples (Table 1), that were able to identify the number of fungal species based on BLAST analysis. These sequences were used for further analysis. The average length of the sequence reads was 440 bp.

**Table 1:** Numbers of sequence reads generated by the GS-FLX Titanium sequencer.

Sample	Dandruff status^a^	No. of sequence reads				
		Raw seq.	Seq. with blast hit	(+) strain^b^	(−) strain^b^	No. of reads used for analysis
G1_B1	normal	12353	12257	6552	5705	10735
G1_B2	normal	11238	11132	5353	5779	10029
G1_B3	normal	13473	13418	7144	6274	12119
G3_B6	normal	11281	11222	5787	5435	9614
G4_B4	normal	10280	10183	5593	4590	9092
G5_B5	patient	8373	8344	4496	3848	7589
G5_B7	patient	7813	7782	3869	3913	7034
Total		74811	74338	38794	35544	66212

a “Normal” status is a healthy scalp and a “patient” status is a dandruff-afflicted scalp.

b (+):positive strand; (−): reverse complementary strand.

### Fungus identification by statistical analysis

Statistical analysis found that the three phyla and 11 genera identified by sequencing and BLAST analysis were significantly different in frequency between healthy scalps and dandruff-afflicted scalps (Table 2). At the phylum level, Basidiomycota were isolated from dandruff-afflicted scalps twice as often as from the healthy scalps. Sequences derived from fungi of the genera *Acremonium*, *Coniochaeta*, *Cryptococcus*, *Didymella*, *Rhodotorula*, and uncultured *Ascomycete* were found in greater frequency in libraries generated from health scalp samples. By contrast, *Eupenicillium*, *Filobasidium, Malassezia*, and *Penicillium* were significantly distributed in samples from the dandruff-afflicted scalps (p<0.0001) along with uncultured soil fungus (p = 0.0014).

**Table 2:** Fungal classifications and differences between normal and dandruff-afflicted scalps.

Classification level	Name	% average		Estimate	Standard error	*p*-value
		Normal	Patient			
Phylum	Ascomycota	82.57	61.30	0.2979	0.0093	<.0001
	Basidiomycota	13.12	35.70	−1.0011	0.0178	<.0001
	Uncultured fungus	4.31	3.00	0.3627	0.0413	<.0001
Genus	*Acremonium* spp.	61.77	57.91	0.042	0.0102	<.0001
	*Coniochaeta velutina*	0.49	0.00	5.0654	1.0032	<.0001
	*Cryptococcus* spp.	12.31	0.26	3.8312	0.109	<.0001
	*Didymella* spp.	20.22	0.05	6.0094	0.2503	<.0001
	*Eupenicillium* spp.	0.01	0.04	−1.8498	0.7596	0.0149
	*Filobasidium floriforme*	0.20	33.85	−5.1703	0.1274	<.0001
	*Malassezia* spp.	0.07	1.91	−3.376	0.2218	<.0001
	*Penicillium* spp.	0.05	3.44	−4.3043	0.2599	<.0001
	*Rhodotorula* spp.	0.84	0.05	2.8899	0.2654	<.0001
	Uncultured ascomycete	1.92	0.02	4.3478	0.3559	<.0001
	Uncultured soil fungus	2.14	2.47	−0.1667	0.0521	0.0014

### Phylum Identification of fungal communities

**Figure 1:**
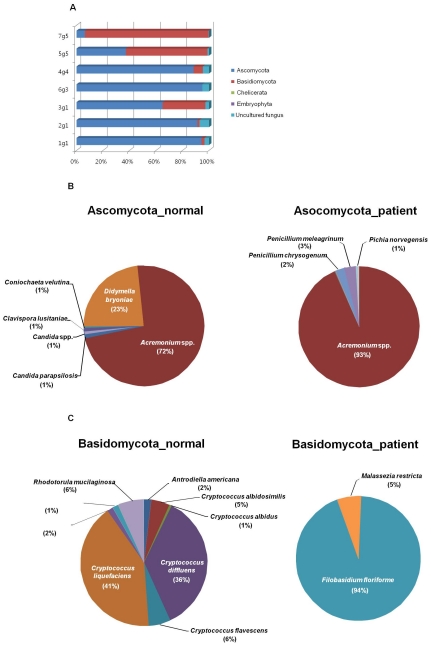
Diagram of mycobiome communities at the phylum level. (A) Distribution of mycobiomes between the normal scalps and the dandruff-afflicted scalps. (B) Distribution of genera belonging to the phylum Ascomycota in healthy and dandruff-afflicted scalps. (C) Distribution of genera belonging to the phylum Basidiomycota in healthy and dandruff-afflicted scalps.

The most dominant fungal phylum in samples from the dandruff-afflicted scalps was Ascomycota consisted of 94.31%, 91.23%, 65.00%, 95.01%, 88.32%, 37.21% and 6.28% of the fungal population of each swab sample, while Basidicomycota consisted of 2.32%, 1.55%, 32.26%, 0.02%, 7.06%, 61.66% and 93.49% of the fungal population of each swab sample (Figure 1A). A total of 53 species of the phylum Ascomycota were detected in the samples overall. Relatively thirty-seven species were identified on healthy scalps and 24 were identified on dandruff-afflicted scalps. Eighteen species of *Basidomycota* were detected on healthy scalps and 11 species were identified on dandruff-afflicted scalps. Among phylum Ascomycota, *Acremonium* spp. was the most dominant fungi on both healthy and dandruff-afflicted scalps.

On healthy scalps, *Acremonium* spp. and *Didymella bryoniae* made up over 95% (72% and 23%, respectively) of the Ascomycota, which is >80% of the fungal community on the healthy scalp compared to just over 60% in patients. In the patient scalp >90% of the Ascomycota are *Acremonium* spp with several *Penicillium* spp., including *P. chrysogenum* and *P. meleagrinum* at (2% and 3%, respectively) (Figure 1B). Among phylum Basidiomycota, populations of Cryptococcus spp. observed on healthy scalps were replaced with Filobasidium (94%) on dandruff-afflicted scalps (Figure 1C).

### Genus identification of fungal communities

**Figure 2:**
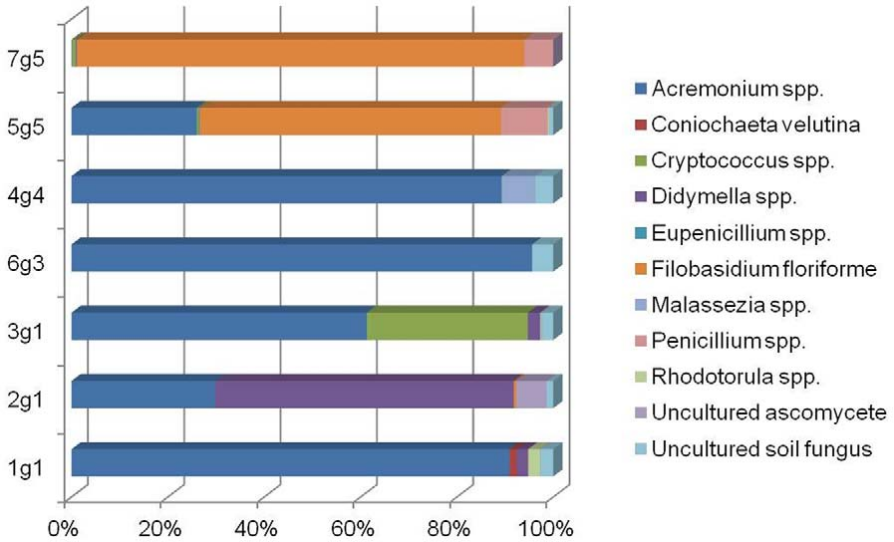
Diagram of the fungal communities at the generic level. Generic frequencies of mycobiomes in individual samples.

A total of 47 culturable and 9 non-culturable genera were identified in at least once sample from both the healthy and dandruff-afflicted scalps. Among them, five to six genera comprised more than 1% of the total fungal community of the grade 1–4 scalps and 2–4 genera had greater than 1% frequency in the grade 5 dandruff-afflicted scalps. In samples from healthy scalps, *Acremonium, Didymella*, and uncultured soil fungus were predominant. Among samples from the each grade scalps sample, *Acremonium* spp. was the major microbes identified, consisting of 91.0%, 61.3%, 95.6 and 89.3% of 1g1, 2g1, 6g3 and 4g4 respectively higher than 2g1, 5g5, and 7g5 (29.8%, 26.0 and 0.1). However, statistical analysis showed that the frequency of *Acremonium* spp.was significantly higher in the healthy scalps than in the dandruff-afflicted scalps (p<0.0001). *Didymella bryoniae* consisted of 62% of fungal community in the 2g1 but they were only isolated below 3% in the 1g1 and 3g1, 1% in the 6g3, 4g4, 5g5 and 7g5. *Filobasidium floriforme* exhibited high frequency, 62.5% and 92.9 in the 5g5, and 7g5 scalps, both of which were graded as having the most severe dandruff condition (Figure 2). Even though the total frequency was lower, *Penicillium* (*P. chrysogenum* and *P. meleagrinum*) had the same tendency as *Filobasidium floriforme*. Among the samples from the healthy scalps, only 3g1 had a high frequency (32%) of genus *Cryptococcus* including *C. albidosiilis* (1.72%), *C. diffluens* (12.81%), and *C. liquefaciens* (14.7%). We found several *Malassezia* spp. in 2g1 and 4g4 scalps, including *M. globosa*, *M. restricta*, *M. samuelsii* and one non-culturable species. *Malassezia* spp. appeared in healthy scalp samples with a frequency of 0.07% and in dandruff-afflicted samples with a frequency of 2%.

## Discussion

We investigated the fungal communities of healthy and dandruff-afflicted human scalps using the GS-FLX Titanium sequencer. Recently, human fungal disease has been recognized as a serious problem and the most common human fungal pathogens belong to the phyla Basidiomycota and Ascomycota, including the major pathogens *Candida albicans*, *Cryptococcus neoformans*, and *Aspergillus fumigates* [17]. Human fungal pathogens are associated with diseases ranging from dandruff and skin colonization to invasive bloodstream infections, which increase morbidity and mortality [18]. This study showed that Ascomycota and Basidiomycota predominate in the fungal community on both healthy and dandruff-afflicted human scalps, although the ratios of abundance of these phyla and their relative species diversity differ between these two scalp categories.

Compared to healthy scalp, the dandruff-afflicted scalp samples showed higher frequencies of the genera *Acremonium* and *Penicillium*, both of the phyla Ascomycota. The genus *Acremonium* currently contains approximately 100 species, and many are recognized as opportunistic pathogens of humans and animals, causing mycetoma, onychomycosis, and hyalohyphomycosis [19]–[21]. Clinical manifestations of hyalohyphomycosis that are caused by *Acremonium* include arthritis, osteomyelitis, peritonitis, endocarditis, pneumonia, cerebritis and subcutaneous infection [19]. However, no clear evidence of association between dandruff with *Acremonium* spp. has been reported. *P. meleagrinum* and *P. chrysogenum* were detected on dandruff-afflicted scalp samples. *Penicillium* is a genus of ascomycetous fungi, and *P. chrysogenum* (*P. notatum*), specifically, produces the antibiotic penicillin. Based on this, we suggest that *Penicillium* may have increased on the dandruff-afflicted scalps because other microbes that cause dandruff, such as *Staphylococcus*, might have increased under the severe condition.

On the dandruff-afflicted scalps, the frequency of the phylum Basidiomycota was also increased. Among the basidiomycetes, *Cryptococcus* species were the most common fungi isolated from healthy scalp. Several species of *Cryptococcus* including *C. liquefaciens*, *C. flavescens*, *C. diffluens*, *C. albidosimilis*, *C. albidus*, *C. magnus*, *C. randhawii*, and *C. oeirensis*, together made up more than 90% of the fungal community on healthy scalps. No pathogenic *Cryptococcus* was detected in the healthy-scalp samples. In addition to the *Cryptococcus* species, *Rhodotorula mucilaginosa* was detected on healthy scalps. *Rhodotorula* (family Cryptococcaceae) is a common airborne fungus found on the skin and in the lungs, urine and feces of humans. Not much is known about the sensitivity of this genus to antifungal agents that are in common clinical use.

In samples from dandruff-afflicted scalps, the Basidiomycota had a different genus composition from that observed on the healthy scalps. Instead of various *Cryptococcus* species, *Filobasidium floriforme* consisted of 94% of the basidiomycete community and *Malassezia* spp. were also increased to constitute 5% of the population. *Filobasidium floriforme* accumulates trehalose, which is widely used as a food ingredient in the United States and Europe [22]. It is also used in cosmetics due to its powerful water-retention properties and in pharmaceuticals due to its ability to preserve tissue and protein [23]. *Malassezia* spp. belong to a sister clade separated from *Cryptococcus* and are not serious human pathogens. *Malassezia* is strongly associated with dandruff, although not all individuals with *Malassezia* on their skin have dandruff [1]. The scalp normally harbors *Malassezia* spp., in addition to many other micro-organisms [24]. During dandruff, the levels of *Malassezia* increase by 1.5–2 times the normal level [8], which is consistent with our results that *Malassezia* increased about two times on dandruff-afflicted scalps over what was observed for the healthy scalps. The increased presence of *Penicillium* and *Filablasidium floriforme* on the dandruff-afflicted scalps were also correlated to samples from the most severe conditions. It is possible that these genera increased to supply the moisture lost when the dandruff became severe and the scalp became too dry.

Our study of the fungal communities on healthy and dandruff-afflicted scalps both confirmed previous findings and provided new information. As expected, we were able to detect *Malassezia* spp. in samples from the dandruff-afflicted scalps and they were more often isolated in the dandruff condition, even though their total frequencies were relatively low. Other than *Malassezia*, we detected several fungal species on the samples from the dandruff-afflicted scalps. More research on the relationships between those fungal species and dandruff might lead to the identification of causative species. Moreover, the preliminary study was planned to compare only fungal population present in both healthy and afflicted scalps and further studies are aimed at both fungal and bacterial level to present large data, understanding the relationships between fungi and other micro-organisms on the scalp. that is important for providing a blueprint for the prevention and treatment of dandruff.

## Materials and Methods

### Ethics statement

Cases of dandruff in Korea that were collected by Department of Dermatology, University Hospital and were confirmed by Department of Microbiology, College of Medicine, Chung-Ang University according to Park *et al.* [25]. All participants gave written informed consent. For all cases, collected samples were analyzed under protocols approved by the Chung-Ang University College of Medicine IRB (Protocol #2010-02-01).

### Sample collection and DNA extraction

**Figure 3:**
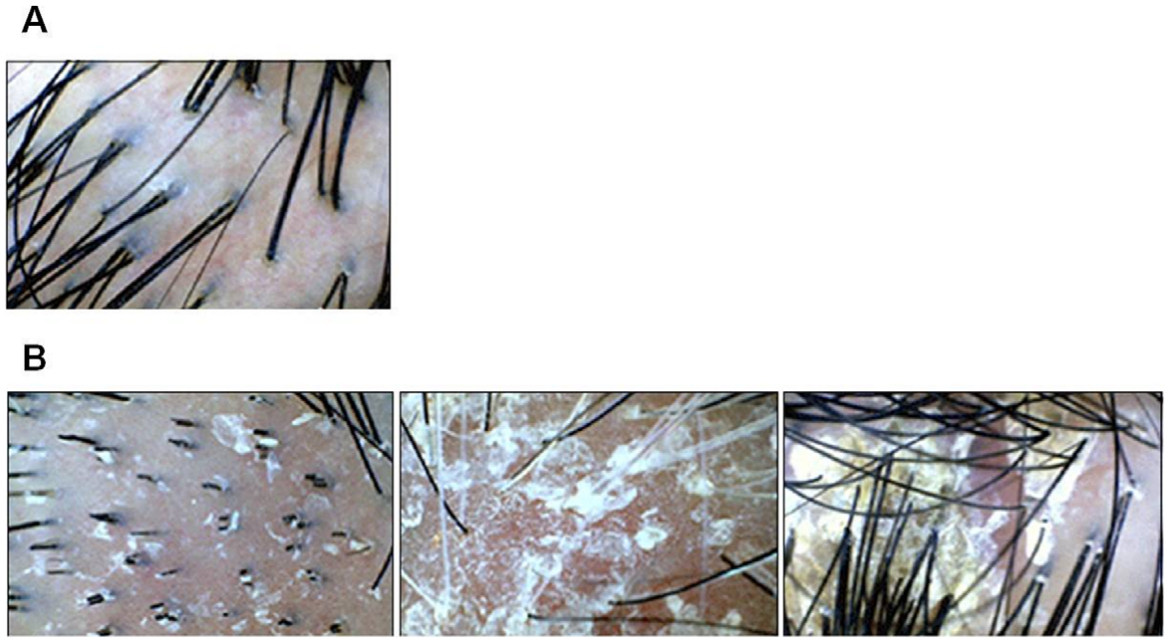
Folliscope images of the sample scalps. (A) Healthy scalp. (B) Dandruff-afflicted scalps.

Human scalp samples were obtained from seven participants who provided written informed consent. The dandruff conditions of the scalps were graded according to Park *et al.* [25] (Figure 3). The samples fell into four grades (g1, g3, g4, and g5) and were further categorized into healthy and dandruff-afflicted groups. Three samples (labeled 1g1, 2g1, and 3g1) were considered to be from healthy scalps with unnoticeable mild or no dandruff present. The other four samples (6g3, 4g4, 5g5 and 7g5) were from heavily dandruff-afflicted scalps. Sample collection was obtained after review by the IRB at Chung-Ang University and University Hospitals. Each sample was obtained in a DNA-free clean room by rubbing the scalp using two sterile cotton swabs soaked in ST solution (0.15 M NaCl with 0.1%Tween 20). The tip of each swab was aseptically cut from the handle and placed into a microcentrifuge tube containing ST solution. The sample tubes were centrifuged for 5 min and the swab tips were then removed from the tube. A negative control was also prepared using cotton swabs that did not contact scalps to detect possible contamination. DNA extraction of the samples was performed using the cetyltrimethylammonium bromide method [26]. DNA was dissolved in sterile water and quantified using the Infinite 200 NanoQuant (Tecan, Switzerland).

### PCR amplification of 26S rRNA gene

We amplified the D1/D2 region of the 26S rRNA gene using NL1 (5′-GCATATCAATAAGCGGAGGAAAAG 3′) and NL4 (5′-GGTCCGTGTTTCAAGACGG 3′) [27] primers to investigate fungal distribution in the samples. For 454 pyrosequencing, 10 base-multiplex identifier (MID) sequences were added to the 5′ ends of both primers. PCR reactions were performed in a 25 µl final volume of reaction mixture containing 5–25 ng of genomic DNA, 0.4 mM of each primer, 0.2 mM dNTPs (Takara), 1.5 mM MgCl_2_ (Invitrogen), 2.0 U Hot Start Taq polymerase (Takara) and 1.0× reaction buffer (Invitrogen). PCR amplification was carried out using the GeneAmp PCR system 9700 (Applied Biosystem) with the following thermal conditions: initial denaturation for 5 min at 94°C; 30 cycles of 30 s at 94°C, 30 s at 55°C and 1 min at 72°C; a final extension for 10 min at 72°C. The PCR products were separated on a 1.2% agarose gel (Seakem LE; FMC Bioproducts) and visualized using a UV transilluminator after ethidium bromide staining.

### 454 pyrosequencing

Equal amounts of PCR products were taken from the seven samples and pooled for 454 sequencing analysis. The pooled DNA was prepared as described in the GS-FLX Titanium sequencing manual and loaded onto a PicoTiter Plate. The plate was inserted into the PicoTiter Plate device and pyrosequencing was performed.

### Sequence analysis

After the sequencing run was completed, the sequence file (.fna) and quality file (.qual) were retrieved by MID groups. For sequence reads in the forward direction, reads with sizes of <100 bases excluded from further analysis. Reverse sequence reads with sizes of >300 were used for analysis. BLAST analysis was performed to identify fungi in the populations.

### Statistical analysis

We applied factor analysis in order to extract meaningful variables from the total observed set of variables. First we identified principal components with the usual standard, which is over 70% of the cumulative variance. We then calculated the component matrix of the chosen principal components and deleted variables whose factor loadings were below 0.6. We then considered the Generalized Linear Model (GLM) using the final set of chosen variables. The following equation (a) represents the GLM, *g*(*μ*) = *α*+*βx* (a) where *t* is the total number of occurrences, *x* = 
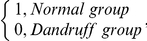
, and *g*(*μ*) = log(*μ*/*t*) in the pooled group of samples from dandruff-afflicted and healthy scalps. The parameter beta is tested at a 5% significance level by (a).
